# Predictors and prognoses of Willisian collateral failure during mechanical thrombectomy

**DOI:** 10.1038/s41598-020-77946-7

**Published:** 2020-11-30

**Authors:** Seong-Joon Lee, Yang-Ha Hwang, Ji Man Hong, Jin Wook Choi, Dong-Hun Kang, Yong-Won Kim, Yong-Sun Kim, Jeong-Ho Hong, Joonsang Yoo, Chang-Hyun Kim, Bruce Ovbiagele, Andrew Demchuk, Sung-Il Sohn, Jin Soo Lee

**Affiliations:** 1 Department of Neurology, Ajou University School of Medicine, Ajou University Medical Center, 164, World cup-ro, Yeongtong-gu, Suwon, Gyeonggi-do 16499 South Korea; 2 Department of Neurology, School of Medicine, Kyungpook National University, Daegu, South Korea; 3 Department of Radiology, Ajou University School of Medicine, Ajou University Medical Center, Suwon, South Korea; 4 Department of Neurosurgery, School of Medicine, Kyungpook National University, Daegu, South Korea; 5 Department of Radiology, School of Medicine, Kyungpook National University, Daegu, South Korea; 6 Department of Neurology, Dongsan Medical Center, Brain Research Institute, Keimyung University School of Medicine, 56 Dalseong-ro Joong-gu, Daegu, 41931 Republic of Korea; 7 Department of Neurology, National Health Insurance Service Ilsan Hospital, Goyang, Korea; 8 Department of Neurosurgery, Keimyung University Dongsan Medical Center, Daegu, Republic of Korea; 9 Department of Neurology, University of California, San Franscisco, USA; 10 Department of Clinical Neurosciences and Radiology, Hotchkiss Brain Institute, University of Calgary, Calgary, AB Canada

**Keywords:** Neurological disorders, Neurology, Diseases, Neurological disorders, Cerebrovascular disorders

## Abstract

During mechanical thrombectomy in the anterior cerebral circulation, thrombus embolization resulting in Willisian collateral failure may lead to critical stroke outcomes due to a shutdown of leptomeningeal collaterals. We hypothesized that the outcomes of dynamic Willisian collateral failure (DWF), induced during mechanical thrombectomy, would be associated with grave outcomes. We evaluated this hypothesis in consecutive patients, between January 2011 and May 2016, who underwent mechanical thrombectomy for anterior circulation occlusions, with an onset-to-puncture of 24 h. Patients with initial Willisian collateral failure (IWF) were identified first, with remaining patients classified into the DWF and Willisian collateral sparing (WCS) groups. Comparative and multivariable analyses were performed to predict *grave* outcomes (3-month modified Rankin Scale score of 5–6). Among 567 patients, 37 were in the IWF group, 38 in the DWF group, and 492 in the WCS group. Compared to the WCS and DWF groups, the IWF group had a higher baseline National Institute of Health Stroke Scale score and lower Alberta Stroke Program Early CT Score. The prevalence of *grave* outcomes was similarly high in the IWF (48.6%) and DWF (47.4%) groups, but lower in the WCS group (22.0%; *p* < 0.001). IWF and DWF were independent risk factors for a *grave* outcome.

## Introduction

Mechanical thrombectomy for cerebral infarcts resulting from large vessel occlusions (LVOs) is a very effective treatment^1^, which aims to salvage cells within the penumbra and arrest infarct growth. However, there can be potential complications, such as access site complications, hemorrhagic complications, device-related complications, and embolization of thrombus resulting in new ischemic events^2^. Embolization of thrombus occurs in up to 6% of mechanical procedures, resulting in infarct growth^2^. In these cases, failure of Willisian collaterals is thought to result in fast infarct growth and poor clinical outcomes.

Willisian collateral failure often occurs at the initial stroke event (initial Willisian collateral failure [IWF]) due to complex ICA terminus occlusions (CTO)^3^. It can occur in an internal carotid artery (ICA) terminus occlusion when thrombus occludes the A2 trunk or distal branches of the anterior cerebral artery (ACA) and/or the P2 segment or distal branches of the posterior cerebral artery (PCA) in combination with occlusion in the middle cerebral artery (MCA). CTO can also functionally occur without involving the ICA terminus when embolus lodges itself in neighboring major intracranial vessels concomitantly (e.g., respective occlusions of the A2 trunk of the ACA and M1 segments of the MCA). Such Willisian collateral failure may also occur during thrombectomy due to thrombus manipulation or distal embolization. Theoretically, such an event would result in near-complete shutdown of leptomeningeal collaterals in the affected hemisphere, potentially harming the patient. The frequency and influence of such dynamic Willisian collateral failure (DWF) have not been previously reported.

Thus, in this study, our aim was to evaluate the effect of DWF on *grave* clinical outcomes for an LVO of the anterior cerebral circulation and to evaluate factors associated with *good* clinical outcomes after DWF. Identification of these factors could guide a future bailout plan during DWF to protect against increasing stroke severity.

## Methods

### Patient enrollment and evaluation

Patients were retrospectively identified from the Acute Stroke due to Intracranial Atherosclerotic occlusion and Neurointervention—Korean Retrospective (ASIAN KR) registry^4,5^. Between January 2011 and May 2016, 720 patients who underwent endovascular treatment (EVT) for an acute ischemic stroke caused by intracranial and/or extracranial LVO were identified. From these patients, those who underwent EVT for an LVO of the anterior circulation (intracranial ICA and M1 segment of the MCA) within 24 h of stroke onset were included in this study. The type of EVT procedure performed was at the discretion of the treating physician, with direct aspiration and stent retrieval primarily used in most cases^6–9^.

A 3-month modified Rankin Scale (mRS) score of 0–2 or no change, compared to the premorbid mRS score, was classified as a *good* outcome, with a 3-month mRS score of 5–6 classified as a *grave* outcome. After de-identification and blinding of clinical data, assessment of laboratory images was performed to ensure consistent grading and to eliminate the possibility of bias.

On preprocedural diffusion-weighted images (DWIs), the infarct volume was evaluated by manually outlining the DWI hyperintense lesions using the NordicICE semi-automated software (NordicNeuroLab, Bergen, Norway) (J.W.C.). Successful reperfusion was defined as a modified Treatment In Cerebral Ischemia (mTICI) grade of 2b (more than 50% reperfusion of target downstream territory) or 3 (complete reperfusion)^10^. Post-procedural hemorrhagic transformations were classified based on the criteria defined by the European Cooperative Acute Stroke Study as none, hemorrhagic infarct type 1 or 2, or parenchymal hematoma type 1 or 2^11^. The post-procedural subarachnoid hemorrhage was graded using the modified Fisher grading system^12^. A parenchymal hematoma type 2 or Fisher grade 3 or 4 subarachnoid hemorrhage was considered as serious hemorrhagic complications.

Conventional leptomeningeal collateral circulation was assessed using single-phase or dynamic computed tomography angiography (CTA). Two different collateral grading systems were applied: the Miteff scoring system^13^ for CTA in general and the modified ASITN/SIR system for dynamic CTA, according to the criteria proposed by Higashida et al.^14^ Collateral circulation was subsequently categorized as poor (Miteff grades 1 and 2; ASITN/SIR grades 0, 1, and 2) or good (Miteff grade 3; ASITN/SIR grades 3 and 4). Collateral circulation could not be graded by CTA in cases of intracranial ICA occlusions with sparing of the ICA bifurcation due to the presence of antegrade flow, rather than a retrograde flow from collaterals.

### Statement of ethics

The data collection protocol was approved by the institutional review board of each participating hospital and implemented in accordance with the ethical standards of the 1964 Declaration of Helsinki and its later amendments. The need for written informed consent was waived given the retrospective design of the study. All data included in this study are available upon reasonable request to the corresponding author.

### Definition of DWF and patient grouping

**Figure 1:**
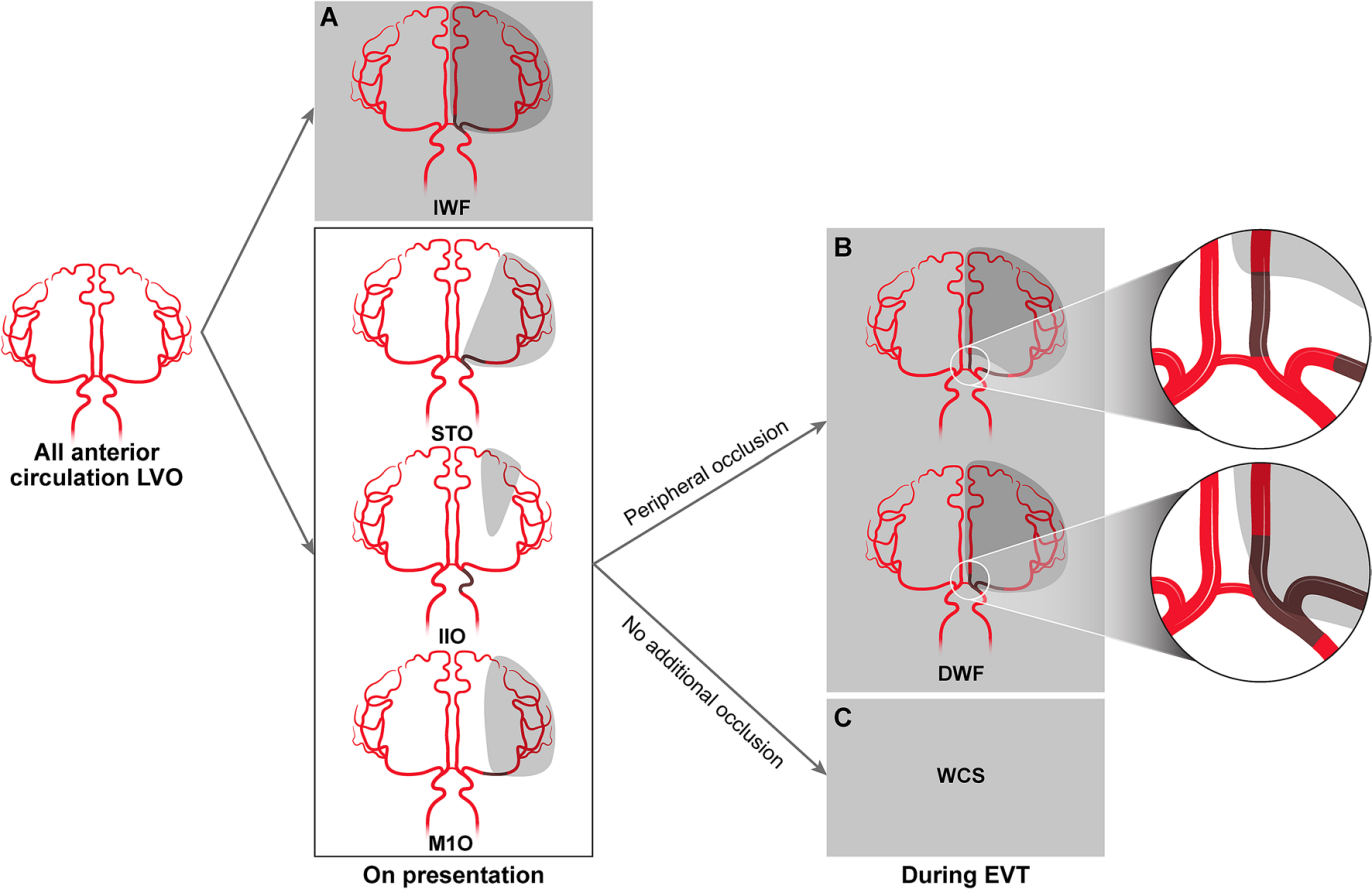
A flowchart of patient grouping based on the dynamic Willisian collateral status. All patients with an intracranial anterior circulation LVO were first classified as (**A**) IWF if they presented with an acute occlusion of the ICA terminus or M1 segment of the MCA in combination with occlusion of the ipsilateral A2 segment or occlusion of the ipsilateral fetal-PCA. Also, an ICA terminus occlusion with agenesis or severe hypoplasia of the contralateral A1 segment or occlusion of the contralateral ICA occlusion were classified as IWF. In the rest anterior circulation occlusions with preserved Willisian collateral flow, patients were classified as (**B**) DWF if a new occlusion of the A2 segment of the ACA, fetal PCA, or both occurred without resolution of the original M1 or intracranial ICA occlusion during EVT. The classification of DWF was made regardless of the final reperfusion outcomes, which is why the term ‘dynamic’ was used. Patients who did not experience DWF or IWF were classified as (**C**) WCS. DWF, dynamic Willisian collateral failure; EVT, endovascular treatment; ICA, internal carotid artery; IIO, isolated intracranial internal carotid artery occlusion; IWF, initial Willisian collateral failure; LVO, large vessel occlusion; MCA, middle cerebral artery; M1O, middle cerebral artery M1 occlusion; PCA; posterior cerebral artery; STO, simple internal carotid artery terminus occlusion; WCS, Willisian collateral sparing.

Changes in occlusion were evaluated by procedural angiography, where all changes that occurred from after the initial CTA or magnetic resonance angiography (MRA) up till the end of the EVT procedure (S.J.L.) were included. Following this analysis, patients were classified into three groups, namely the IWF, DWF, and Willisian collateral sparing (WCS) groups (Fig. 1). IWF was first classified according to pre-procedure CTA or MRA if a CTO pattern or its equivalent was observed^3^. In brief, an acute ICA terminus occlusion or an M1 occlusion combined with: (1) occlusion of the ipsilateral A2 or more distal segment; (2) occlusion of the fetal-type ipsilateral posterior cerebral artery, was considered IWF. In addition, an ICA terminus occlusion with: (1) insufficient contralateral Willisian collateral blood supply via the anterior communicating artery due to contralateral agenesis of the A1 segment, or; (2) contralateral ICA occlusion, was classified as IWF. Patients who presented with (1) conventional M1 occlusion (M1O), (2) simple ICA T occlusions (STO), or (3) isolated ICA I occlusion (IIO) with sparing of the ICA bifurcation, were considered to be spared of IWF. In these patients, DWF was defined as an embolization during EVT resulting in occlusion of another major intracranial artery without resolution of the initial occlusion. In this DWF type, a new occlusion of the ACA A2, fetal PCA, or both occurred in combination with a functional M1 occlusion, causing a near complete shutdown of blood flow. DWF was classified regardless of final occlusion status. Patients who did not present with IWF or did not experience DWF were classified in the WCS group. Various patterns could be included in the WCS group, as long as there was no Willisian collateral failure; absence of embolization, distal embolization within the same arterial bed, and occlusion of another territory after complete reperfusion of the initial territory were all classified WCS.

### Statistical analysis

Clinical characteristics, imaging and endovascular therapy findings, and outcomes were compared between the IWF, DWF, and WCS groups. Univariate analysis was performed using the chi-squared (χ^2^) test for categorical variables. For continuous variables, an analysis of variance with a Bonferroni post-hoc analysis was used for comparison between three groups, with Student’s t-test for comparison between two groups. The effect of Willisian collateral failure on the risk of *grave* functional outcomes was further analyzed using a multiple logistic regression analysis, adjusting for clinically significant variables. In the DWF subgroup, patients with good and poor functional outcomes were compared. To identify procedural factors associated with a *good* outcome in this subgroup, a multiple logistic regression analysis was performed on variables presumed to be clinically significant.

Data are presented as the mean ± standard deviation, median [interquartile range], or number (%) as appropriate for the data type and distribution. A *p*-value < 0.05 was considered statistically significant. All statistical analyses were performed using IBM SPSS Statistics (version 22; IBM Corp., Armonk, NY, USA).

## Results

### Occlusion changes during the procedure

Among the 720 patients identified in the ASIAN KR database, 567 presented with primary occlusion of the intracranial ICA and MCA M1, within 24 h of symptom onset. The distribution of the type of occlusion was as follows: 37 (6.5%) IWF, 38 (6.7%) DWF, and 492 (86.8%) WCS. Among patients in the DWF group, 37 had a new occlusion into ACA, and 1 into PCA.

### Effect of Willisian collateral failure on functional outcomes and its predictors

**Figure 2:**
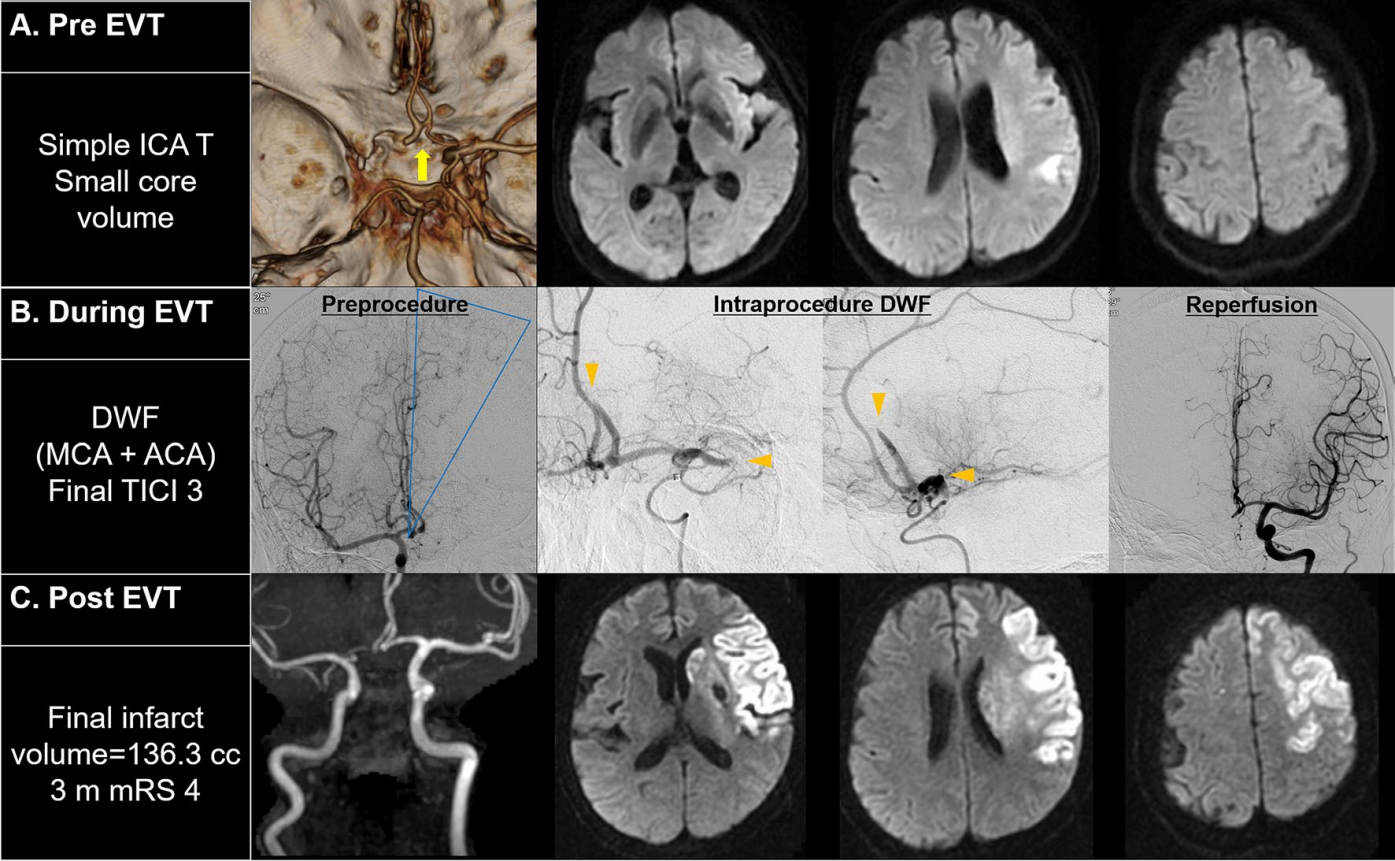
An example of dynamic Willisian collateral failure during endovascular therapy. (**A**) Preprocedural imaging reveals preservation of the Willisian collaterals by the anterior communicating artery (yellow arrow) and shows a small infarct volume. (**B**) Angiography of the contralateral ICA, before the procedure, confirms the patency of the leptomeningeal collaterals of the ipsilateral anterior cerebral artery (blue triangle). During the procedure, DWF occurs, and a new embolization into the A2 segment of the ACA (orange arrowheads) abruptly resulting in a near-complete leptomeningeal collateral shutdown. (**C**) Despite complete reperfusion achieved on final angiography, a large final infarct volume was induced, due to DWF, which was associated with poor outcomes. ACA, anterior cerebral artery; DWF, dynamic Willisian collateral failure; EVT, endovascular treatment; ICA, internal carotid artery; MCA, middle cerebral artery; mRS, modified Rankin Scale; mTICI, modified thrombolysis in cerebral ischemia.

Comparisons between the IWF, DWF, and WCS groups are reported in Table 1. The National Institutes of Health Stroke Scale (NIHSS) score was higher and the Alberta Stroke Program Early CT Score (ASPECTS) lower in the IWF group than in the WCS group (IWF versus WCS, *p* < 0.005, post hoc Bonferroni test), with no difference in scores between the DWF and WCS groups. The reperfusion procedure time was the longest for the DWF group (DWF vs. WCS, *p* = 0.007, post hoc Bonferroni test). The rate of successful recanalization was slightly lower for the DWF group (70.3% vs. 60.5% vs. 77.6%, *p* = 0.041), while the rate of complete TICI grade 3 reperfusion was significantly lower for the DWF group (2.6%) compared to the rate for the IWF (18.9%) and WCS (33.3%) groups (*p* < 0.001 for overall TICI grades). The rate of a *grave* outcome was higher in the IWF (48.6%) and DWF (47.4%) groups than in the WCS (22.0%) group (*p* < 0.001). Of note, patients with an IWF presented with a large baseline infarct volume (51 [20–210] ml) and ended up with a large final infarct volume (109 [15–229] ml), while patients in the DWF group presented with a smaller baseline infarct volume (11 [5–43] ml) but a large final infarct volume (97 [23–167] ml). Patients in the WCS group presented with a small baseline infarct volume (11 [5–31] ml) and a smaller final infarct volume (28 [11–85] ml) (baseline infarct volume, *p* < 0.001; and final infarct volume, *p* < 0.001). The rate of significant hemorrhagic complications was higher in the IWF group than in the DWF and WCS groups (35.1% vs. 10.5% and 9.6%, respectively, *p* < 0.001). On multivariable analysis, both IWF (odds ratio [OR]: 2.44, 95% confidence interval [CI]: [1.15–5.20], *p* = 0.020) and DWF (OR: 2.82, 95% CI: [1.34–5.95], *p* = 0.007) were associated with a *grave* outcome, with the OR adjusted by age, sex, premorbid mRS, admission NIHSS score, onset-to-puncture time, intravenous thrombolytic administration, and final successful reperfusion (Table 2. model 1). When baseline DWI infarct volume was further included as a covariate, only DWF (OR: 3.21, 95% CI: [1.38–7.49], *p* < 0.001) was significantly associated with grave outcomes (Table 2. model 2). An example of a DWF resulting in a large infarct volume, regardless of the final reperfusion status, and poor outcomes is depicted in Fig. 2.

**Table 1:** Clinical characteristics, imaging and endovascular therapy findings, and outcomes, according to the dynamic Willisian collateral status in acute anterior circulation occlusion.

	IWF (n = 37)	DWF (n = 38)	WCS (n = 492)	*p* value
Sex, male	17 (45.9%)	21 (55.3%)	269 (54.7%)	0.584
Age, years	71 ± 12	68 ± 12	67 ± 13	0.172
Diabetes mellitus	12 (32.4%)	13 (34.2%)	128 (26.0%)	0.407
Hypertension	21 (55.3%)	28 (75.7%)	300 (61.0%)	0.148
Atrial fibrillation	22 (59.5%)	20 (52.6%)	242 (49.2%)	0.459
Initial NIHSS	19.0 [17.0–22.0]	18.5 [14.0–22.0]	16.0 [13.0–20.0]	0.001^a^
ASPECTS	5.0 [2.0–8.0]	6.0 [3.5–8.0]	7.0 [5.0–9.0]	0.001^a^
**Primary occlusion location**				< 0.001
CTO	37 (100.0%)	–	–	
Simple ICA T	–	23 (60.5%)	144 (29.3%)	
IIO	–	5 (13.2%)	32 (6.5%)	
M1O	–	10 (26.3%)	316 (64.2%)	
Tandem occlusion	5 (13.5%)	4 (10.5%)	49 (10.0%)	0.788
**CTA collateral (n = 290)**	n = 22	n = 19	n = 285	0.880
Good	6 (27.3%)	6 (31.6%)	75 (26.3%)	
Poor	16 (72.7%)	13 (68.4%)	210 (73.7%)	
Intravenous tPA	22 (59.5%)	19 (50.0%)	262 (53.3%)	0.695
Onset-to-puncture, min	315 ± 252	347 ± 222	332 ± 231	0.832
Procedure time, min	83 ± 51	94 ± 55	71 ± 43	0.004^b^
**Primary intracranial modality**				0.236
Stent retrieval	15 (40.5%)	9 (23.7%)	158 (32.1%)	
Direct aspiration	21 (56.8%)	29 (76.3%)	307 (602.4%)	
Others	1 (2.7%)	0 (0.0%)	27 (5.5%)	
Balloon guide catheter used	28 (75.7%)	24 (63.2%)	349 (70.9%)	0.473
**Reperfusion outcomes**				< 0.001
mTICI 0	1 (2.7%)	1 (2.6%)	41 (8.3%)	
mTICI 1	1 (2.7%)	2 (5.3%)	9 (1.8%)	
mTICI 2A	9 (24.3%)	12 (31.6%)	60 (12.2%)	
mTICI 2B	19 (51.4%)	22 (57.9%)	218 (44.3%)	
mTICI 3	7 (18.9%)	1 (2.6%)	164 (33.3%)	
Successful reperfusion	26 (70.3%)	23 (60.5%)	382 (77.6%)	0.041
Baseline DWI (ml, n = 484)	51 [20–210]	11 [5–43]	11 [5–31]	< 0.001^c^
Follow-up DWI (ml, n = 427)	109 [15–229]	97 [23–167]	28 [11–85]	< 0.001^d^
PH2 or SAH 3–4	13 (35.1%)	4 (10.5%)	47 (9.6%)	< 0.001
Favorable outcomes	11 (29.7%)	12 (31.6%)	265 (54.0%)	0.001
Grave outcomes	18 (48.6%)	18 (47.4%)	108 (22.0%)	< 0.001

The data are presented as the mean ± standard deviation, number (%), or median [interquartile range], as appropriate.

ASPECTS, Alberta Stroke Program Early CT scores; CTO, complex internal carotid artery terminus occlusion; DWF, dynamic Willisian collateral failure; IIO, isolated intracranial internal carotid artery occlusion; IQR, interquartile range; IWF, initial Willisian collateral failure; M1O, middle cerebral artery M1 occlusion; mTICI, Modified thrombolysis In Cerebral Ischemia; NIHSS, National Institute of Health Stroke Scale; PH2, parenchymal hematoma type 2; SAH 3–4, subarachnoid hemorrhage Fisher grade 3–4; tPA, tissue plasminogen activator; WCS, Willisian collateral sparing.

^a^IWF versus WCS, *p* < 0.005, post hoc Bonferroni test.

^b^DWF versus WCS, *p* = 0.007, post hoc Bonferroni test.

^c^IWF versus DWF & WCS, *p* < 0.005, post-hoc Bonferroni test.

^d^WCS versus IWF & DWF, *p* < 0.05, post-hoc Bonferroni test.

**Table 2:** Logistic regression models predictive of *grave* outcomes, according to the Willisian collateral status.

Groups	Odds ratio (95% CI)	*p* value
*Model 1 (total population)*^a^		
**Willisian collateral status**		0.003
WCS	Reference	
IWF	2.44 [1.15–5.20]	0.020
DWF	2.82 [1.34–5.95]	0.007
*Model 2 (DWI volume adjusted, N = 484)*^b^		
**Willisian collateral status**		0.022
WCS	Reference	
IWF	1.49 [0.54–4.09]	0.439
DWF	3.21 [1.38–7.49]	< 0.001

DWI, diffusion weighted images; DWF, dynamic Willisian collateral failure; IWF, initial Willisian collateral failure; WCS, Willisian collateral sparing.

^a^Adjusted by age, sex, premorbid modified Rankin score, admission National Institute of Health Stroke Scale score, onset-to-puncture time, intravenous administration of tissue plasminogen activator, and final successful reperfusion.

^b^Adjusted by baseline DWI volume, age, sex, premorbid modified Rankin score, admission National Institute of Health Stroke Scale score, onset-to-puncture time, intravenous administration of tissue plasminogen activator, and final successful reperfusion.

### Predictors of DWF

As for predicting factors for DWF, a simple ICA T occlusion or isolated intracranial ICA occlusion as a primary occlusion type was the only predictor—among the 530 patients without IWF, DWF occurred in 23 of 167 patients (13.8%) with STO, 5 of 37 patients (13.5%) with an IIO, and 10 of 326 patients (3.1%) with an M1 occlusion (*p* < 0.001). Intravenous administration of tissue plasminogen activator, presence of tandem occlusions, type of reperfusion procedure (such a balloon guide catheter), and primary reperfusion modality were not predictive DWF.

### Factors associated with a *good* outcome in the DWF group

Within the DWF subgroup, the comparison of characteristics of patients that achieved good and poor outcomes is shown in Table 3. Patients with good outcomes despite DWF, compared to those with a poor outcome, had a lower rate of diabetes mellitus (8.3% vs. 46.2%, respectively, *p* = 0.022), atrial fibrillation (25.0% vs. 65.4%, respectively, *p* = 0.020), and higher rate of tandem ICA occlusions (25.0% vs. 3.8%, respectively, *p* = 0.048). Follow-up DWI volume was also much smaller in the good compared to poor outcome group (26 [9–95] ml vs. 77 [147–184] ml, respectively, *p* = 0.006). While the rate of successful reperfusion was only marginally significant at 83.3% for patients with a good outcome compared to 50.0% for those with a poor outcome (*p* = 0.051), the rate of successful reperfusion of newly embolized territory was significantly higher in the good outcome group (91.7%) than in the poor outcome group (30.8%, *p* < 0.001), with shorter procedure time (53 ± 27 min vs. 113 ± 55 min, respectively, *p* < 0.001). In the multivariable analysis, total procedure time (inverse correlation, OR: 0.94, 05% CI: [0.90–0.99], *p* = 0.012) and reperfusion of the newly embolized vascular territory (OR: 21.42, 95% CI: [1.20–383.30], *p* = 0.037) were independent predictors of a *good* outcome, along with age and successful reperfusion as covariates (Table 4).

**Table 3:** Comparison of characteristics of patients that achieved good outcomes in patients which DWF occurred during procedure.

	Good outcome (N = 12)	Poor outcome (N = 26)	*p* value
Sex, male	5 (41.7%)	16 (61.5%)	0.252
Age, years	66 ± 9	69 ± 13	0.448
Diabetes mellitus	1 (8.3%)	12 (46.2%)	0.022
Hypertension	7 (58.3%)	14 (53.8%)	0.796
Atrial fibrillation	3 (25.0%)	17 (65.4%)	0.020
Initial NIHSS	16.5 [14.25–21.75]	19.0 [14.0–22.25]	0.839
ASPECTS	6.0 [4.5–7.5]	7.0 [3.0–8.0]	0.793
**Primary occlusion location**			0.722
Simple ICA T	7 (58.3%)	16 (61.5%)	
IIO	1 (8.3%)	4 (15.4%)	
M1O	4 (33.3%)	6 (23.1%)	
Tandem occlusion	3 (25.0%)	1 (3.8%)	0.048
Intravenous tPA	7 (58.3%)	12 (46.2%)	0.485
Onset-to-puncture, min	291 ± 121	373 ± 254	0.188
Procedure time, min	53 ± 27	113 ± 55	< 0.001
**Primary intracranial modality**			0.342
Stent retrieval	4 (33.3%)	5 (19.2%)	
Direct aspiration	8 (66.7%)	21 (80.8%)	
Balloon guide catheter used	9 (75.0%)	15 (57.7%)	0.304
**DWF type**			0.136
MCA + ACA	11 (91.7%)	26 (100.0%)	
MCA + PCA	1 (8.3%)	0 (0.0%)	
**Reperfusion outcomes**			0.145
mTICI 0	0 (0.0%)	1 (3.8%)	
mTICI 1	1 (8.3%)	1 (3.8%)	
mTICI 2A	1 (8.3%)	11 (42.3%)	
mTICI 2B	9 (75.0%)	13 (50.0%)	
mTICI 3	1 (8.3%)	0 (0.0%)	
Successful reperfusion	10 (83.3%)	13 (50.0%)	0.051
Reperfusion of newly embolized territory	11 (91.7%)	8 (30.8%)	< 0.001
Baseline DWI (ml, n = 484)	11 [8–53]	12 [4–46]	0.678
Follow-up DWI (ml, n = 427)	26 [9–95]	77 [147–184]	0.006
PH2 or SAH 3–4	0 (0.0%)	4 (15.4%)	0.151

The data are presented as the mean ± standard deviation, number (%), or median [interquartile range], as appropriate.

ASPECTS, Alberta Stroke Program Early CT scores; DWF, dynamic Willisian collateral failure; DWI, diffusion weighted images; IIO, isolated intracranial internal carotid artery occlusion; IQR, interquartile range; M1O, middle cerebral artery M1 occlusion; mTICI, modified thrombolysis In Cerebral Ischemia; NIHSS, National Institute of Health Stroke Scale; PH2, parenchymal hematoma type 2; SAH 3–4, subarachnoid hemorrhage Fisher grade 3–4tPA, tissue plasminogen activator; WCS, Willisian collateral sparing.

**Table 4:** Logistic regression model predictive of *good* outcomes in the DWF subgroup.

	Odds ratio (95% CI)	*p*
Procedure time	0.94 [0.90–0.99]	0.012
Reperfusion of newly embolized territory	21.42 [1.20–383.30]	0.037
≥ mTICI 2B reperfusion	5.66 [0.21–149.58]	0.299
Age, years	0.94 [0.83–1.08]	0.381

CI, confidence interval; DWF, dynamic Willisian collateral failure; mTICI, Modified Thrombolysis in Cerebral Ischemia.

## Discussion

The principal finding of this study is that clinical outcomes of anterior circulation LVOs were strongly influenced by the Willisian collaterals, even when it occurs during the reperfusion process. DWF occurs in approximately 7% of all anterior circulation LVOs and were mostly associated with embolization of thrombus to the ACA. DWF resulted in grave outcomes comparable to IWF, which is likely due to the rapid growth of infarct volume. Good outcomes despite DWF was associated with reduced procedure time and reperfusion of the newly embolized vascular territory.

Unlike previous studies, we measured presence of embolic complications by whether Willisian collateral failure occurred or not. While some studies have evaluated the effect of embolization to the ACA^15^ or distal embolization in the same territory^16^, the mechanistic rationale was not based on the patency of Willisian collaterals. Our findings indicate that DWF has a critical effect on outcomes in contrast to other embolic events that do not, such as distal embolizations^16^. Simultaneous occlusion of the MCA and ACA or PCA results in near-total impairment of collateral flow to the affected hemisphere, resulting in a rapid infarct growth rapidly exceeding the threshold for good clinical outcomes even with successful reperfusion. Moreover, in the presence of DWF, a grade 3 mTICI reperfusion appears to be difficult to accomplish, which is another factor associated with poor observed outcomes.

When DWF occurred during the procedure, shorter procedure time and reperfusion of the newly embolized vascular territory, which mostly occurred in the main branch of the ACA, were predictors of *good* clinical outcomes. The specific importance of reperfusion of the ACA has been previously reported. In a retrospective review of 105 recanalization procedures for M1 occlusions, ACA occlusion occurred in 11.4% of the cases, with the resultant ACA infarcts limiting post-stroke motor recovery^15^. Endovascular recanalization of major ACA branches reduced the burden of infarcts with no adverse events^15^. Generally, reperfusion of the ACA is avoided because of its smaller arterial caliber and the risk of procedural complications. However, according to the limited clinical evidence available, the recanalization rate is reported to be high and periprocedural complications are rare^17,18^. Considering these factors, reperfusion of the ACA may be pursued in feasible patients as a bailout therapy for DWF.

In our dataset, we could not find any preprocedural variables that predicted DWF, other than the location of the initial occlusion, and no specific reperfusion modality could prevent such failure from occurring. DWF more commonly occurred in STO and IIO types of occlusion than M1 occlusions, likely due to the large thrombus burden. Factors such as intravenous administration of tissue plasminogen activator, which was previously reported to cause a pre-interventional change of occlusion site resulting in worsening of perfusion (due to an effect on collaterals)^19^, or the use of balloon guide catheters, known to reduce distal embolization^20^, was not predictive of DWF. However, taking into consideration the grave consequences of DWF, the authors advocate the use of the balloon guide catheters^21^ or other methods to improve first-pass effect^22^.

The outcome of IWF in this study is consistent with those in previous studies, even with modern thrombectomy procedures. The outcome of carotid terminus occlusions was once considered to be grave due to the low rate of reperfusion and high rate of hemorrhagic transformations^23^. However, advances in thrombectomy techniques, including the use of longer stent retrievers^24^, large-bore aspiration catheters^25^, and balloon guide catheters^26^, as well as the use of combination modalities^27^, have led to a high rate of reperfusion for carotid terminus occlusions. With these improvements in EVT outcomes, ICA terminus occlusions can now usually be categorized along with MCA M1 occlusions under the term anterior circulation LVO. However, there is still a population where outcomes are universally grave despite best EVT. These patients may be candidates for combining EVT with future therapeutic advances, such as neuroprotection or hypothermia^28^.

The limitations of our study need to be mentioned. First, MR imaging was not performed in a small number of patients, with infarct growth only measured in patients who underwent follow-up MR imaging. However, follow-up MR imaging could not be performed in deceased patients or in patients with a medically *grave* condition due to a large infarct. Accordingly, the increase in infarct size in the DWF group was likely to have been under-estimated than over-estimated. Second, predictors of good outcome despite DWF were only evaluated in a small number of patients, possibly resulting in lower predictive power. In this regard, the corresponding results should be confirmed in larger prospective registry data. Third, CTA collateral grades were not included in the multivariable analysis for two reasons; conventional leptomeningeal collateral scores cannot be applied to IIO and the grade was available in only about half of the total study sample. Thus, the added benefit of evaluating Willisian collateral failure over conventional leptomeningeal collateral grading could not be shown in this study. Fourth, the results of this study could not identify the factors associated with an increased risk of DWF, except for the location of the initial occlusion. It remains to be determined if advances in reperfusion techniques, such as the use of the Solumbra technique^29^ or others, may reduce the rate of DWF.

## Conclusion

In EVT of LVOs of the anterior circulation, DWF most commonly occurred by concurrent occlusion of the previously uninvolved ACA and was significantly associated with *grave* outcomes along with IWF. As good outcomes despite DWF are associated with total procedure time and reperfusion of the newly embolized vascular territory, rapid reperfusion of the ACA may be considered when feasible.
